# Role of Prolactin and Growth Hormone on Thymus Physiology

**DOI:** 10.1155/1998/89782

**Published:** 1998

**Authors:** Valéria De Mello-Coelho, Wilson Savino, Marie-Catherine Postel-Vinay, Mireille Dardenne

**Affiliations:** 1 CNRS URA 1461 Université Paris V Hôpital Necker 161, rue de Sèvres, Cedex 15 Paris 75743 France; 2 lNSERM U 344 Faculté de medecine Necker-Enfants Malades Paris France; 3 Laboratory on Thymus Research Department of Immunology Institute Oswaldo Cruz Foundation Oswaldo Cruz Rio de Janeiro Brazil

**Keywords:** Thymus, thymic hormone, thymic epithelial cells, thymocyte differentiation, prolactin, growth hormone

## Abstract

Intrathymic T-cell differentiation is under the control of the thymic microenvironment, which acts on maturing thymocytes via membrane as well as soluble products. Increasing data show that this process can be modulated by classical hormones, as exemplified herein by prolactin (PRL) and growth hormone (GH), largely secreted by the pituitary gland.

Both PRL and GH stimulate the secretion of thymulin, a thymic hormone produced by thymic epithelial cells. Conversely, low levels of circulating thymulin parallel hypopituitary states. Interestingly, the enhancing effects of GH on thymulin seem to be mediated by insulinlike growth factor (IGF-1) since they can be abrogated with anti-IGF-1 or anti-IGF-l-receptor antibodies. The influence of PRL and GH on the thymic epithelium is pleiotropic: PRL enhances *in vivo* the expression of high-molecular-weight cytokeratins and stimulates *in vitro* TEC proliferation, an effect that is shared by GH and IGF-1.

Differentiating T cells are also targets for the intrathymic action of PRL and GH. *In vivo* inoculation of a rat pituitary cell line into old rats results in restoration of the thymus, including differentiation of CD4^-^CD8^-^ thymocytes into CD4^+^CD8^+^ cells. Furthermore, PRL may regulate the maintenance of thymocyte viability during the double-positive stage of thymocyte differentiation.

Injections of GH into aging mice increase total thymocyte numbers and the percentage of CD3-bearing cells, as well as the Concanavalin-A mitogenic response and IL-6 production by thymocytes.  Interestingly, similar findings are observed in animals treated with IGF-1. Lastly, the thymic hypoplasia observed in dwarf mice can be reversed with GH treatment.

In keeping with the data summarized earlier is the detection of receptors for PRL and GH on both thymocytes and thymic epithelial cells. Importantly, recent studies indicate that both cell types can produce PRL and GH intrathymically. Similarly, production of IGF-1 and expression of a corresponding receptor has also been demonstrated.

In conclusion, these data strongly indicate that the thymus is physiologically under control of pituitary hormones PRL and GH. In addition to the classical endocrine pathway, paracrine and autocrine circuits are probably implicated in such control.


*Developmental Immunology*, 1998, Vol. 6, pp. 317–323
Reprints available directly from the publisher
Photocopying permitted by license only

© 1998 OPA (Overseas Publishers Association)
N.V. Published by license under the
Harwood Academic Publishers imprint,
part of the Gordon and Breach Publishing Group
Printed in Malaysia

### Role of Prolactin and Growth Hormone on Thymus Physiology

VALÉRIA DE MELLO-COELHO[b], WILSON SAVINO[c], MARIE-CATHERINE POSTEL-VINAY[b] and MIREILLE DARDENNE[a*†]

[a]*CNRS URA 1461, Université Paris V, Hôpital Necker, Paris, France;* [b]*INSERM U 344, Faculté de medecine Necker-Enfants Malades, Paris, France;* [c]*Laboratory on Thymus Research, Department of Immunology, Institute Oswaldo Cruz, Foundation Oswaldo Cruz, Rio de Janeiro, Brazil*

(*Received 15 August 1996; In final form 4 August 1997; Accepted 10 August 1997*)

Intrathymic T-cell differentiation is under the control of the thymic microenvironment, which acts on maturing thymocytes via membrane as well as soluble products. Increasing data show that this process can be modulated by classical hormones, as exemplified herein by prolactin (PRL) and growth hormone (GH), largely secreted by the pituitary gland.
Both PRL and GH stimulate the secretion of thymulin, a thymic hormone produced by thymic epithelial cells. Conversely, low levels of circulating thymulin parallel hypopituitary states. Interestingly, the enhancing effects of GH on thymulin seem to be mediated by insulinlike growth factor 1 (IGF-1) since they can be abrogated with anti-IGF-1 or anti-IGF-1-receptor antibodies.
The influence of PRL and GH on the thymic epithelium is pleiotropic: PRL enhances *in vivo* the expression of high-molecular-weight cytokeratins and stimulates *in vitro* TEC proliferation, an effect that is shared by GH and IGF-1.
Differentiating T cells are also targets for the intrathymic action of PRL and GH. *In vivo* inoculation of a rat pituitary cell line into old rats results in restoration of the thymus, including differentiation of $CD4^-CD8^-$ thymocytes into $CD4^+CD8^+$ cells. Furthermore, PRL may regulate the maintenance of thymocyte viability during the double-positive stage of thymocyte differentiation.
Injections of GH into aging mice increase total thymocyte numbers and the percentage of CD3-bearing cells, as well as the Concanavalin-A mitogenic response and IL-6 production by thymocytes. Interestingly, similar findings are observed in animals treated with IGF-1. Lastly, the thymic hypoplasia observed in dwarf mice can be reversed with GH treatment.
In keeping with the data summarized earlier is the detection of receptors for PRL and GH on both thymocytes and thymic epithelial cells. Importantly, recent studies indicate that both cell types can produce PRL and GH intrathymically. Similarly, production of IGF-1 and expression of a corresponding receptor has also been demonstrated.
In conclusion, these data strongly indicate that the thymus is physiologically under control of pituitary hormones PRL and GH. In addition to the classical endocrine pathway, paracrine and autocrine circuits are probably implicated in such control.

*Keywords:* Thymus, thymic hormone, thymic epithelial cells, thymocyte differentiation, prolactin, growth hormone

*Corresponding author, Tel.: (33) (1) 44-495392, Fax: (33) (1) 44-490676, E-mail: dardenne@infobiogen.fr.
†Present address: CNRS URA-1461, Hôpital Necker, 161, rue de Sèvres, 75743, Paris, Cedex 15, France.

### INTRODUCTION

Bone-marrow-derived prothymocytes migrate into the thymus where they undergo a complex process of differentiation, largely dependent on interactions with the thymic microenvironment, a tridimensional cellular network formed by epithelial cells, macrophages, dendritic cells, and fibroblasts (reviewed by van Ewijk, 1991; Boyd et al., 1993). Such interactions are transient, involving sessile elements such as microenvironmental cells, and migrating components, the differentiating thymocytes. Along with migration and differentiation, most thymocytes are negatively selected (the majority being deleted by apoptosis), whereas some appear to be positively selected, rescued from death, eventually yielding the vast majority of the so-called T-cell repertoire. One key cellular interaction defining positive or negative selection involves the TCR/CD3 complex expressed by differentiating thymocytes with class I or class II products of the major histocompatibility complex (MHC) present on the microenvironmental cell membranes, associated with the endogenous peptide to be recognized (Ashton-Rickardt and Tonegawa, 1994).

In addition, the thymic microenvironment influences thymocyte migration/differentiation via other types of interactions. For example, thymic epithelial cells (TECs) release soluble polypeptides such as thymic hormones and various cytokines (see reviews by Safieh-Garabedian et al., 1992; Kendall and Stebbings, 1994; Boyd et al., 1993) that can modulate thymocyte behavior. In this respect, thymulin, one chemically defined thymic hormone is able to induce the expression of CD90—the Thy-1 marker—(see review by Bach, 1983) that has been defined as an adhesion molecule able to transduce signals for the intrathymic production of cytokines during thymocyte differentiation (Tentori et al. 1988; He et al., 1991). In addition to other immune functions (see reviews by Bach, 1983; Safieh-Garabedian et al., 1992), thymulin acts on neuroendocrine circuits, modulating the secretion of various hypothalamic and pituitary hormones (see reviews by Dardenne and Savino, 1996). Lastly, TEC/thymocyte interactions can also be mediated via classical adhesion molecules as well as extracellular matrix (ECM) ligands and receptors (reviewed by Patel and Haynes, 1993; Savino et al., 1993).

A further important concept concerns the influence of extrinsic factors (endogenous and exogenous) on the thymic microenvironment. In particular, recent studies point to a neuroendocrine control of the thymus (see review by Dardenne and Savino, 1994). In this respect, we will discuss herein the effects of prolactin (PRL) and growth hormone (GH) on various aspects of the thymic microenvironment and T-cell differentiation. Moreover, the intrathymic production of these hormones as well as the expression of corresponding receptors by thymic cells will be analyzed, in an attempt to provide a molecular basis for autocrine, paracrine, and endocrine pathways that may be involved in the action of both PRL and GH on thymus physiology.

### THYMIC ENDOCRINE FUNCTION IS INFLUENCED BY PITUITARY HORMONE STATUS

The first demonstration that pituitary hormones can modulate endocrine function came from studies in dwarf mice that exhibit a precocious age-dependent decay of thymulin serum levels (Pelletier et al., 1976). In keeping with this finding, low thymulin levels are found in hypophysectomized rats and dwarf humans (see reviews by Dardenne and Savino, 1990, 1992; Mocheggiani et al., 1994). Conversely, adenopituitary hyperactivity states, including prolactinoma and acromegaly, are paralleled by increased levels of serum thymulin (Timsit et al., 1989, 1992). Additionally, *in vivo* injections of PRL or GH in respectively young or old mice and rats, enhance thymulin secretion (Dardenne et al., 1989; Goya et al., 1992). Lastly, both hormones stimulate thymulin secretion by cultured TEC (Dardenne et al., 1989; Timsit et al., 1992). Interestingly, the enhancing effect of GH on thymulin secretion appears to be mediated by insulinlike growth factor 1 (IGF-1) since GH-induced stimulatory effects can be prevented *in vitro* by treating TEC cultures with antibodies specific for IGF-1 or IGF-1 receptor (Timsit et al., 1992). Accordingly, IGF-1 alone stimulates thymulin production by cultured TEC. Moreover, there is a positive correlation between serum levels of thymulin and IGF-1 in acromegalic patients (Timsit et al., 1992).

### PITUITARY HORMONES PLEIOTROPICALLY INFLUENCE THE THYMIC EPITHELIUM

In addition to the influence of PRL and GH on thymic endocrine function, other aspects of TEC physiology can be modulated by these hormones, thus characterizing their pleiotropic action on the thymic epithelium. For example, PRL is able to enhance *in vivo* the expression of high-molecular-weight cytokeratins that are restrictly found in the medulla of thymic lobules (Dardenne et al., 1989). Additionally, PRL stimulates *in vitro* TEC proliferation (Dardenne et al., 1989), an effect that is shared by GH and IGF-1 (Timsit et al., 1992). More recently, augmentation of ECM expression was seen, not only in cultures of TEC lines, but also in primary cultures of epithelial cells derived from isolation of thymic nurse lymphoepithelial complexes (de Mello-Coelho et al., 1997). Together, these data point to the notion that both PRL and GH can modulate genes committed to distinct biological activities of the thymic epithelium.

### CONTROL OF THYMOCYTE DIFFERENTIATION BY PROLACTIN AND GROWTH HORMONE

In addition to the effects on microenvironmental cells, differentiating T cells are targets for the intrathymic action of PRL and GH. In this respect, it is striking that *in vivo* inoculation of GH3 cells (a rat pituitary cell line able to produce GH and PRL) into old rats results in restoration of the thymus to the histological pattern found in young animals (Kelley et al., 1986). Moreover, in old animals, this procedure promotes differentiation of $CD4^-CD8^-$ thymocytes into $CD4^+CD8^+$ cells (Li et al., 1992). Furthermore, a decrease of double-positive cells was demonstrated, associated with a diminution of thymocyte viability in cell suspensions of neonatal thymuses treated with anti-mouse PRL antibodies, able to neutralize exogenous PRL. In this study, an increase in $CD4^-CD8^-CD25^+$ cells was observed, suggesting that PRL may regulate the maintenance of thymocyte viability during the double-positive stage of thymocyte differentiation, and could be a relevant signal to rescue double-positive cells from apoptosis (Gaufo and Diamond, 1996). Accordingly, an antiapoptotic effect of this hormone was demonstrated on the Nb2 rat thymic lymphoma cell line (La Voie and Witorsch, 1995). Interestingly, it was shown that PRL induces the gene expression of cyclins D2 and D3 that regulate the beginning of Nb2 cell proliferation (Hosokawa et al., 1994). Lastly, the possibility that PRL influences the shaping of T-cell repertoire is suggested, since it can switch the expression of TCR chains in Nb2 cells (Hosokawa et al., 1996).

A series of *in vivo* experiments also evidenced significant changes in thymocyte differentiation under GH influence. Injections of this hormone into aging mice increase total thymocyte numbers and the percentage of CD3-bearing cells (Knyszynski et al., 1992). In addition, Con-A mitogenic response as well as IL-6 production by thymocytes are enhanced in GH-treated aging animals (Goya et al., 1992). Interestingly, cyclosporin-A-induced thymic atrophy is restored by *in vivo* treatment with recombinant GH or IGF-1 (Beschorner et al., 1991). Lastly, IGF-1 is able to induce repopulation of the atrophic thymus from diabetic rats and mice (Binz et al., 1990; Bergerot et al., 1995).

The role of GH on thymus development is also stressed by the findings obtained with GH-deficient dwarf mice. In these animals, besides the precocious decline in thymulin serum values (Pelletier et al., 1976), there is a progressive thymic hypoplasia with decreased numbers of CD4/8 double-positive thymocytes. Importantly, such defects can be largely restored by long treatment with GH (Murphy et al., 1992). It is clear from the data discussed before that a variety of intrathymic changes take place following PRL or GH stimulation, further characterizing the pleiotropic role of these hormones on the thymus.

### EXPRESSION OF PROLACTIN AND GROWTH HORMONE RECEPTORS IN THE THYMUS

To bring further support to the data obtained in both experimental models and humans evidencing a role of PRL and GH on thymus physiology, it was obviously necessary to demonstrate receptors for such molecules on thymic cells. Both PRL and GH receptors belong to the haematopoietin-receptor family (that also includes receptors for various cytokines), which appears to be derived from a common ancestral gene that underwent multiple duplications and modifications (see review by Finidori and Kelly, 1995).

With regard to the PRL receptor, two main forms have been characterized in murine tissues, probably resulting from alternative splicing of a primary transcript (reviewed by Kelly et al., 1993). Both forms of the receptor were reported in thymic epithelial cells and differentiating thymocytes, as ascertained by distinct methodological approaches (Dardenne et al., 1991; Gagnerault et al., 1993; Nagano and Kelly, 1994; Gunes and Mastro, 1996). Additionally, anti-PRL receptor antibodies can act as agonists of the natural ligand, thus mimicking PRL in enhancing TEC proliferation and thymulin secretion (Dardenne et al., 1991). It is also noteworthy that the Concanavalin-A-induced mitogenic response of thymocytes is paralleled by an augmentation of PRL receptor density on thymocyte membranes, suggesting that PRL may be related to intrathymic T-cell activation (Gagnerault et al., 1993). Together, these data indicate that PRL receptors in thymic cells are functional.

Intrathymic expression of GH receptor has been demonstrated by different methodological approaches. In cultured thymic epithelial cells, such expression was demonstrated by direct ligand binding and further Scatchard analysis (Ban et al., 1991). Also, *in situ* hybridization studies revealed a positive signal for GH receptor throughout the thymic cortex as well as medullary TEC (Mertani et al., 1995). More recently, this finding was confirmed using cytofluorimetry and RT-PCR (manuscript in preparation). Expression of GH receptor has been demonstrated in thymocytes as well. By using biotinylated GH in tricolor cytofluorimetric studies performed in short-term cultured murine thymocytes, the GH receptor was detected in 20-30% of murine thymocytes, comprising distinct differentiation stages, namely, the immature $CD4^{-}CD8^{-}$ and $CD4^{+}CD8^{+}$ cells, as well as single-positive thymocytes for either CD4 or CD8 marker (Gagnerault et al., 1996). Interestingly, the percentages of GH receptor-bearing thymocytes were transiently increased after Con-A-induced activation.

### INTRATHYMIC PRODUCTION OF PROLACTIN AND GROWTH HORMONE

Extrapituitary sources of PRL and GH have been demonstrated in a variety of tissues, including cells of the immune system (see review by Weigent, 1996). Intrathymically, PRL expression was detected at the mRNA transcript level (Touraine et al., 1994; Wu et al., 1996a), and distinct PRL isoforms were seen in Con-A-stimulated murine and human thymocytes (Montgomery et al., 1990, 1992). Interestingly, recent *in situ* hybridization studies revealed PRL mRNA in thymocytes and to a lesser extent in epithelial cells (Wu et al., 1996a).

Growth hormone production has also been evidenced in TEC, as ascertained by *in situ* hybridization and immunocytochemistry (Maggiano et al., 1994). In this study, it was suggested that only a proportion of TEC would be able to constitutively produce GH. By contrast, in a recent study in which the intrathymic production of GH was ascertained by RT-PCR and *in situ* hybridization, a positive signal for GH mRNA was detected in microenvironmental cells and thymocytes (Wu et al., 1996b). Accordingly, GH release was detected in TEC as well as in thymocyte cultures (unpublished), thus supporting the hypothesis that

Thus, in addition to the classical endocrine pathway, paracrine and autocrine circuits are probably implicated in the PRL/GH control of thymus physiology, as proposed in Fig. 1. Dissecting such hypothetical intrathymic circuitry will be certainly relevant for a better understanding of how the organ functions in daily life.

### *Acknowledgments*

The authors thank D. Broneer for an English review. This work was supported with grants from INSERM, CNRS, and CNPq. V. de Mello-Coelho is a recipient of a scholarship from the Brazilian Research Council (CNPq).

### *References*

Ashton-Rickardt P.G. and Tonegawa S. (1994). A differential avidity model for T cell selection. Immunol. Today **15**:362-366.

Bach J.F. (1983). Thymulin (FTS-Zn). Clin. Immunol. Allergy **3**:133-157.

Ban E., Gagnerault M.C., Jammes H., Postel Vinay M.C., Haour F. and Dardenne M. (1991). Specific binding sites for growth hormone in cultured mouse thymic epithelial cells. Life Sci. **48**:2141-2148.

Bergerot I., Fabien N., Maguer V. and Thivolet C. (1995). Insulin-like growth factor-1 (IGF-1) protects NOD mice from insulitis and diabetes. *Immunology* **102**:335-340.

Beschorner N.E., Divic J., Pulido H., Yao X., Kenworthy P. and Bruce G. (1991). Enhancement of thymic recovery after cyclosporine by recombinant human growth hormone and insulin-like growth factor I. *Transplantation* **52**:879-884.

Binz K., Joller P., Froesch P., Binz H., Zapf J. and Froesch E.R. (1990). Repopulation of atrophied thymus in diabetic rats by insulin-like growth factor-I. Proc. Natl. Acad. Sci. USA **87**:3690-3694.

Boyd R.L., Tucek C.L., Godfrey D.I., Izon D.J., Wilson T.J., Davidson N.J., Bean A.G.B., Ladyman H.M., Ritter M.A. and Hugo P. (1993). The thymic microenvironment. Immunol. Today **14**:445-459.

Dardenne M., Savino W., Gagnerault M.C., Itoh T. and Bach J.F. (1989). Neuroendocrine control of thymic hormonal production. I. Prolactin stimulates in vivo and in vitro the production of thymulin by human and murine thymic epithelial cells. Endocrinology **125**:3-12.

Dardenne M., Kelly P.A., Bach J.F. and Savino W. (1991). Identification and functional activity of prolactin receptors in thymic epithelial cells. Proc. Natl. Acad. Sci. USA **88**:9700-9704.

Dardenne M. and Savino W. (1990). Neuroendocrine control of the thymic epithelium: Modulation of thymic endocrine function, cytokeratin expression and cell proliferation, by hormones and neuropeptides. Prog. NeuroEndocrineImmunol. **3**:18-25.

Dardenne M. and Savino W. (1992). Neuroendocrine circuits controlling the physiology of the thymic epithelium. Ann. N. Y. Acad. Sci. **650**:85-90.

Dardenne M. and Savino W. (1994). Neuroendocrine control of thymus physiology by peptidic hormones and neuropeptides. Immunol. Today **15**:518-523.

Dardenne M. and Savino W. (1996). The interdependence of the endocrine and immune systems. Adv. Neuroimmunol. **6**:297–307

Delhase M., Vergani P., Malur A., Hooghe-Peters E.L. and Hooghe R.J. (1993). The transcription factor Pit-1/GHF-1 is expressed in hematopoietic and lymphoid tissues. Eur. J. Immunol. **23**:951-955.

De Mello-Coelho V., Villa-Verde D.M.S., Dardenne M. and Savino W. (1997). Pituitary hormones modulate extracellular matrix-mediated interactions between thymocytes and thymic epithelial cells. J. Neuroimmunol. **76**:39–49

Finidori J. and Kelly P.A. (1995). Cytokine receptor signalling through two novel families of transducer molecules: Janus kinases, signal transducers and activators of transcription. J. Endocrinol. **147**:11-23.

Gagnerault M.C., Postel-Vinay M.C. and Dardenne M. (1996). Expression of growth hormone receptors in murine lymphoid cells analyzed by flow cytofluorimetry. Endocrinology **137**:1719-1726.

Gagnerault M.C., Touraine P., Savino W., Kelly P. and Dardenne M. (1993). Expression of prolactin receptor on murine lymphoid cells in normal and autoimmune conditions. J. Immunol. **151**:1-9.

Gaufo G.O. and Diamond M.C. (1996). Prolactin increases CD4/CD8 cell ratio in thymus-grafted congenitally athymic nude mice. Proc. Natl. Acad. Sci. USA **93**:4165-4169.

Geenen V., Achour I., Robert F., Vandersmissen E., Sodoyez J., Defresne M.P., Boniver J., Lefebvre P.J. and Franchimont P. (1993). Evidence that insulin-like growth factor 2 (IGF-2) is the dominant thymic peptide of the insulin superfamily. *Thymus* **21**:115-120.

Goya R.G., Gagnerault M.C., Leite de Moraes M.C., Savino W. and Dardenne M. (1992). In vivo effects of growth hormone on thymus function in aging mice. Brain Behav. Immunity. **6**:341-350.

Gunes H. and Mastro A.M. (1996). Prolactin receptor expression in rat splenocytes and thymocytes from birth to adulthood. Mol. Cell. Endocrinol. **117**:41-52.

He H.T., Naquet P., Caillol D. and Pierres M. (1991). Thy-1 supports adhesion of mouse thymocytes to thymic epithelial cells through $Ca2^{+}$-independent mechanism. J. Exp. Med. **173**:515-518.

Hosokawa Y., Onga T. and Nakashima K. (1994). Induction of D2 and D3 cyclin-encoding genes during promotion of the G1/S transition by prolactin Nb2 cells. Gene **147**:249-252.

Hosokawa Y., Yang M., Kaneko S., Tanaka M. and Nakashima K. (1996). Prolactin induces switching of T-cell receptor gene expression from $\alpha$ to $\gamma$ in rat Nb2 pre-T lymphoma cells. Biochem. Biophys. Res. Communic. **220**:958-962.

Kelley K.W., Brief S., Westly H.J., Novakofki J., Betchel P.J., Simon J. and Walker E.B. (1986). GH3 pituitary adenoma cells reverse thymic aging in rats. Proc. Natl. Acad. Sci. USA **83**:5663-5667.

Kelly P., Ali S., Rozaskis M., Goujon L., Nagano M., Pellegrini I., Gould D., Djiane D., Edery M., Finidori J. and Postel-Vinay M.C. (1993). The growth hormone/prolactin receptor family. Recent Prog. Horm. Res. **18**:123-163.

Kendall M.D. and Stebbings R.J. (1994). The endocrine thymus. Endocrine J. **2**:333-339.

Knyszynski A., Adler-Kunin S. and Globerson A. (1992). Effects of growth hormone on thymocyte development from progenitor cells in the bone marrow. Brain Behav. Immunity. **6**:327-340.

La Voie H.A. and Witorsch R.J. (1995). Investigation of intracellular signals mediating the anti-apoptotic action of prolactin in Nb2 lymphoma cells. Proc. Soc. Exp. Biol. Med. **209**:257-269.

Li Y.M., Brunke D.L., Dantzer R. and Kelley K.W. (1992). Pituitary epithelial cells implants reverse the accumulation of CD4-CD8- lymphocytes in thymus gland of aged rats. Endocrinology **130**:2703-2709.

Maggiano N., Piantelli M., Ricci R., Larocca L., Capelli A. and Ranelletti F.O. (1994). Detection of growth hormone-producing cells in human thymus by immunohistochemistry and non-radioactive *in situ* hybridization. J. Histochem. Cytochem. **42**:1349-1354.

Mertani H.C., Delehaye-Zervas M.C., Martini J.F., Postel-Vinay M.C. and Morel G. (1995). Localization of growth hormone receptor messenger RNA in human thymocytes. Endocrine **3**:135-142.

Mocchegiani E., Paolucci P., Balsamo A., Cacciari E. and Fabris N. (1994). Influence of growth hormone on thymic endocrine activity in humans. Hormone Res. **33**:7-12.

Montgomery D.W., Lefevre J., Ulrich E.D., Steiner L.L., Adamson C. and Zukoski C.F. (1990). Identification of prolactin-like synthesized by normal murine cells. Endocrinology **127**:2601-2603.

Montgomery D.W., Shen G.K., Ulrich E.D., Steiner L.L., Parrish P.R. and Zukoski C.F. (1992). Human thymocytes express a prolactin-like messenger ribonucleic acid and synthesize bioactive prolactin-like proteins. Endocrinology **131**:3019-3026.

Murphy W.J., Durum S.K. and Longo D.L. (1992). Role of neuroendocrine hormones in murine T cell development. Growth hormones exerts thymopoietic effects in vivo. J. Immunol. **149**:3851-3857.

Nagano M. and Kelly P.A. (1994). Tissue distribution and regulation of the rat prolactin receptor gene expression: quantitative analysis by polymerase chain reaction. J. Biol. Chem. **269**:13337-13345.

Patel D.D. and Haynes B.F. (1993). Cell adhesion molecules involved in intrathymic T cell development. Semin. Immunol. **5**:282-292.

Pelletier M., Montplaisir S., Dardenne M. and Bach J.F. (1976). Thymic hormone activity and spontaneous autoimmunity in dwarf mice and their littermates. *Immunology* **30**:783-788.

Safieh-Garabedian B., Kendall M.D., Khamashta M.A. and Hughes G.R.V. (1992). Thymulin and its role in immunomodulation. J Autoimmunity **5**:547-555.

Savino W., Villa-Verde D.M.S. and Lannes Vieira J. (1993). Extracellular matrix proteins in intrathymic T cell migration and differentiation? Immunol. Today **4**:158-161.

Tentori I., Pardoll D.M., Zuniga-Pflucker J.C., Hui J., Paul W.E., Bluestone J.A. and Kruisbeek A.M. (1988). Proliferation and production of IL-2 and B cell stimulating factor 1/IL-4 in early fetal thymocytes by activation through Thy-1 and CD3. *J. Immunol.* **140**:1089-1094.

Timsit J., Safieh B., Gagnerault M.C., Savino W., Lobetzki J., Bach J.F. and Dardenne M. (1989). Augmentation des taux circulants de thymuline au cours de l'hyperprolactinemie et de l'acromegalie. C. R. Acad. Sci. Paris **310** (Serie III):1-13.

Timsit J., Savino W., Safieh B., Chanson P., Gagnerault M.C., Bach J.F. and Dardenne M. (1992). Growth hormone and insulin-like growth factor 1 stimulates hormonal function and proliferation of thymic epithelial cells. J. Clin. Endocrinol. Metabol. **75**:1251-1260.

Touraine P., Leite-de-Moraes M.C., Dardenne M. and Kelly P. (1994). Expression of short and long forms of prolactin receptor in murine lymphoid tissues. Mol. Cell. Endocrinol. **104**:183-190.

Van Ewijk W. (1991). T-cell differentiation is influenced by thymic microenvironments. Ann. Rev. Immunol. **9**:591-615.

Weigent D.A. (1996). Immunoregulatory properties of growth hormone and prolactin. Pharmacol. Ther. **69**:237-257.

Wu H., Devi R. and Mamarkey W.B. (1996a). Localization of prolactin messenger ribonucleic acid in the human immune system. Endocrinology **137**:349-353.

Wu H., Devi R. and Mamarkey W.B. (1996b). Localization of growth hormone messenger ribonucleic acid in the human immune system—A clinical research center study. J. Clin. Endocrinol. Metabol. **81**:1278-1282.

Yamada M., Hato F., Kinoshita Y., Tominaga K. and Tsuji Y. (1994). The indirect participation of growth hormone in the thymocyte proliferation system. Cell. Mol. Biol. Noisy le grand **40**:111-121.

